# Targeting CAR to the Peptide-MHC Complex Reveals Distinct Signaling Compared to That of TCR in a Jurkat T Cell Model

**DOI:** 10.3390/cancers13040867

**Published:** 2021-02-18

**Authors:** Ling Wu, Joanna Brzostek, Shvetha Sankaran, Qianru Wei, Jiawei Yap, Triscilla Y.Y. Tan, Junyun Lai, Paul A. MacAry, Nicholas R. J. Gascoigne

**Affiliations:** 1 Translational Immunology Research Programme, Yong Loo Lin School of Medicine, National University of Singapore, Singapore 119228, Singapore; ling.wu@u.nus.edu (L.W.); joanna.brzostek@biologie.uni-freiburg.de (J.B.); shvetha_sankaran@gis.a-star.edu.sg (S.S.); micyapj@nus.edu.sg (J.Y.); junyunlai@u.nus.edu (J.L.); micpam@nus.edu.sg (P.A.M.); 2 Department of Microbiology and Immunology, Yong Loo Lin School of Medicine, National University of Singapore, 5 Science Drive 2, Singapore 117545, Singapore; qiw4003@med.cornell.edu (Q.W.); triscilla.tan@u.nus.edu (T.Y.Y.T.); 3 Translational Cancer Research Programme, Yong Loo Lin School of Medicine, National University of Singapore, Singapore 119228, Singapore; 4 Life Sciences Institute, National University of Singapore, Singapore 117456, Singapore

**Keywords:** T cell receptor, chimeric antigen receptor (CAR) T cell (CAR-T), signal transduction, CD8 coreceptor, signaling kinetics, oligomerization

## Abstract

**Simple Summary:**

Chimeric antigen receptors (CARs) redirect T cells without the need for major histocompatibility complex (MHC) restriction. CARs are designed based on T cell receptor (TCR) signaling and the recognition specificities of antibodies. This technology has achieved great clinical success in combatting cancers. Despite these successes, the mechanism of CAR signaling in the T cell and how this can impact function is not fully understood. To enhance our understanding and to identify the characteristics of CAR signaling, we designed a CAR to target a peptide-MHC complex, similar to the TCR. This allowed us to compare CAR and TCR head-to-head, such that novel traits of CAR signaling could be discovered. We found that CAR has distinct signaling characteristics compared to TCR, including the molecules that facilitate signal transduction. These findings offer explanations for the clinical behavior of CAR T cells (CAR-T) therapy and avenues to optimize the technology.

**Abstract:**

Chimeric antigen receptor T cells (CAR-T) utilize T cell receptor (TCR) signaling cascades and the recognition functions of antibodies. This allows T cells, normally restricted by the major histocompatibility complex (MHC), to be redirected to target cells by their surface antigens, such as tumor associated antigens (TAAs). CAR-T technology has achieved significant successes in treatment of certain cancers, primarily liquid cancers. Nonetheless, many challenges hinder development of this therapy, such as cytokine release syndrome (CRS) and the efficacy of CAR-T treatments for solid tumors. These challenges show our inadequate understanding of this technology, particularly regarding CAR signaling, which has been less studied. To dissect CAR signaling, we designed a CAR that targets an epitope from latent membrane protein 2 A (LMP2 A) of the Epstein–Barr virus (EBV) presented on HLA*A02:01. Because of this, CAR and TCR signaling can be compared directly, allowing us to study the involvement of other signaling molecules, such as coreceptors. This comparison revealed that CAR was sufficient to bind monomeric antigens due to its high affinity but required oligomeric antigens for its activation. CAR sustained the transduced signal significantly longer, but at a lower magnitude, than did TCR. CD8 coreceptor was recruited to the CAR synapse but played a negligible role in signaling, unlike for TCR signaling. The distinct CAR signaling processes could provide explanations for clinical behavior of CAR-T therapy and suggest ways to improve the technology.

## 1. Introduction

Chimeric antigen receptor T cell (CAR-T) technology takes advantage of the specificity of an antibody (Ab) and the signaling of a T cell receptor (TCR), such that a T cell can be redirected to target cells in a non-major histocompatibility complex (MHC) restricted manner. CAR-T technology has achieved significant clinical success in recent years, with two commercial products available, Kymriah^®^ (tisagenlecleucel; Novartis, Basel, Switzerland) and Yescarta^®^ (axicabtagene ciloleucel; Gilead, Foster City, CA, USA), with many more coming down the pipeline [1,2,3]. A typical CAR construct comprises an extracellular recognition domain, generally a single chain variable fragment (scFv) of an Ab, hinge region, transmembrane, co-stimulatory domain, and signal activation domain. The latter is typically from CD3 z, but other variants, such as CD3 e or CD3 z mutants, have also been shown to have enhanced functionality compared with CD3 z [3]. Each part of the design plays an important role in determining the downstream signaling, but the parameters are not fully understood at this time [3,4].

Understanding CAR signaling is critical to explain the different clinical behavior of each CAR-T therapy and to facilitate rational design of CAR constructs. Taking cytokine release syndrome (CRS) as an example [5], one of the most severe side effects is caused by the strength of CAR signaling while killing the cancer cells. The Gasdermin E (GSDME) signal pathway is activated, leading to pyroptosis of cancer cells and increased expression of damage-associated molecular pattern molecules (DAMPs), which in turn activate macrophages to release CRS-related cytokines [6]. Moreover, CD28-CAR-T and CD137-CAR-T have distinct in vitro and in vivo clinical performance. CD28-CAR-T cells were shown to have a higher cytotoxic activity and antigen sensitivity in vitro and in vivo than did CD137-CAR-T cells [7,8]. The less potent CD137-CAR signaling was later demonstrated to be caused by the recruitment of the negative signaling molecular complex THEMIS-SHP1 [9], which is important in integrating T cell signal strength [10]. Overexpression of LCK countered this negative regulation and led to enhanced CD137-CAR-T therapy [9].

Several studies have been performed to understand intracellular CAR signaling networks [11,12,13]. CAR signaling was found to have a different magnitude and different kinetics of phosphorylation events compared to those of TCR signaling. Some novel protein interactions, such as capsule synthesis 1 domain containing 1 (CASD1), butyrophilin-like 3 (BTNL3), and an additional form of CD3ζ, p21, have been found to be associated with CAR signaling [13]. Nevertheless, the mechanism and impact of these interactions are poorly understood. To better decipher CAR signaling and its mechanism, it is important to compare CAR and TCR head-to-head, such that the divergence of CAR signaling from TCR signaling can be delineated. However, most of the studies thus far have been based on tumor associated antigens (TAAs), such as CD19, that may omit important molecules involved in TCR signaling, such as coreceptors. Therefore, a CAR designed to target the peptide-MHC (pMHC) complex can offer a better comparison between CAR and TCR. Only a few studies using this comparison have been done so far, where CAR was predicted to have a distinct signaling by mathematical modelling, but such a distinct signaling patten has not yet been substantiated [14,15].

To this end, we designed a CAR based on a “TCR-like” Ab that targets a peptide from latent membrane protein 2 A (LMP2 A) protein of the Epstein–Barr virus (EBV) presented by HLA-A*02:01. The affinity of the TCR-like Ab is in the nanomolar range, whereas TCRs are typically micromolar [16]. These affinities are within the normal ranges for Ab and TCR so that they imitate the real situation. In this study, we found that CAR signal activation requires clustering—since the pMHC monomer would not initiate the CAR signaling—and that CAR signaling produces more sustained calcium signaling kinetics compared with TCR. We observed recruitment of the CD8 coreceptor into the CAR signaling synapse, but the CD8 coreceptor did not elevate the CAR signaling, unlike the case with TCR signaling. These findings are important for our understanding of how CAR initiates signaling and how it differs from TCR signaling, and could potentially explain differences in clinical behavior of CAR-T from other adoptive T cell therapies.

## 2. Results

### 2.1. CAR Signal Initiation Requires Clustering

**Figure 1:**
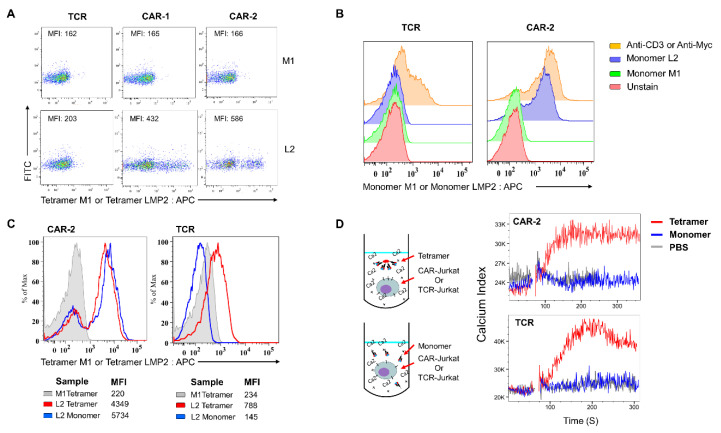
Monomer or tetramer binding and activation to the chimeric antigen receptor (CAR) and T cell receptor (TCR). (**A**) TCR and CAR binding to the specific peptide-major histocompatibility complex (pMHC) tetramer. CAR-1 and CAR-2 refer to first and second generation of CAR design, respectively. L2 and M1 represents the antigenic LMP2 A_426–434_ (CLGGLLTMV) and irrelevant M1_58–66_ (GILGFVFTL) peptide tetramer, respectively. The tetramer was formed by loading monomers with antigen presenting cell (APC)-streptavidin. Overall mean fluorescent intensity is labeled in the graph. (**B**) Monomer binding to CAR and TCR. The monomer binding was detected by using anti-B2 M APC. Anti-CD3-APC and Anti-Myc-APC were used to measure the expression of TCR and CAR on the surface, respectively. (**C**) Comparison of monomer and tetramer binding to CAR and TCR. (**D**) CAR and TCR activation upon monomer and tetramer binding. The schematic graph is shown at the left panel, the tetramer and monomer activation groups contained equal amounts of monomer. The Ca^2+^ index was calculated by the ratio of the mean fluorescence intensity (MFI) of BUV395 to the MFI of Indo-1 (Bv421). The cells were stabilized for 1 to 1.5 min, followed by addition of the tetramer or monomer. The calcium flux was recorded for an additional 3–4 min. All data are representative of at least three independent experiments.

A TCR-like Ab targeting the peptide LMP2 A_426–434_ (“L2”; sequence CLGGLLTMV) from EBV presented by HLA-A*02:01 was utilized to generate first and second generation CARs containing CD28 costimulatory domain [17,18] (Figure S1A). These were stained with pMHC (A*02:01) tetramers. The CAR-1 or CAR-2-transduced Jurkat cells showed strong binding to the L2-tetramer, but no binding to an irrelevant M1-tetramer, which presented M1_58–66_ (GILGFVFTL) from the influenza A virus (Figure 1A). Besides tetramer binding capacity, the CAR protein also showed significant binding to the L2-A*02:01 monomer. This monomer-binding capacity was comparable to the Ab staining of the CAR protein (Figure 1B) and tetramer (Figure 1C). In comparison, TCR showed binding capacity only to the tetramer (Figure 1B,C). Nevertheless, the monomer-binding ability of CAR did not result in activation (Figure 1D). The CAR signal was transduced when the monomers were clustered with the tetramer, as was the case for TCR. However, as seen by Western blotting, the size of CAR-1 and CAR-2 without dithiothreitol (DTT) appeared to be two times bigger than the reduced samples (Figure S1C). This indicates that CAR exists as a covalently linked dimer in the cells.

### 2.2. CAR Transduces a Sustained but Lower Signaling Compared with TCR

**Figure 2:**
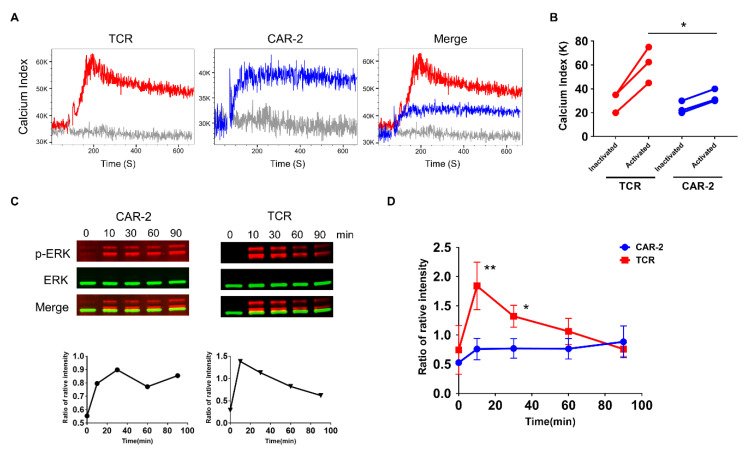
Sustained CAR and TCR signaling kinetics by Ca^2+^ flux and downstream phosphorylation. (**A**) Long-term Ca^2+^ flux of CAR and TCR signaling. The Ca^2+^ flux was recorded for 10 min after the tetramer was added. Calcium index was calculated as the ratio of BUV395/Indo-1 (UV). The negative control, shown in grey, was from after adding PBS. (**B**) Quantification data of TCR and CAR Ca^2+^ flux. Activated data dots indicate the highest value of the Ca^2+^ flux from TCR or CAR samples. (**C**) Phosphorylation of the ERK molecule of CAR and TCR after stimulation at different timepoints. Phosphorylation strength of pERK1/2 was calculated by comparing the intensity of p-ERK to that of total ERK. The two graphs are then grouped to compare their intensity strength; Uncropped Western Blots are available in Figure S3. (**D**) Quantification data of p-ERK of TCR and CAR-Jurkat cells after activation. All data are representative of at least three independent experiments. Student’s *t* test, two-tailed, was used to measure the significance; * *p* < 0.05, ** *p* < 0.01.

A difference in Ca^2+^ signaling kinetics between CAR and TCR was observed (Figure 1D), where CAR signaling reached and sustained a plateau, but TCR signaling waned after reaching a peak. We therefore extended the duration for Ca^2+^ flux detection from 3–4 min to 10 min, to test whether the sustained Ca^2+^ flux would last over a longer period. CAR signaling was more sustained than was TCR signaling (Figure 2A). Moreover, the sustained CAR signaling was not attributable to the costimulatory domain, since the first generation CAR-1 also showed signal transduction in a sustained manner (Figure S2). The phosphorylation of downstream signaling molecules, such as ERK, was also shown to be consistent with the distinct CAR signaling kinetics (Figure 2C). Though more sustained compared with TCR signaling, the magnitude of CAR signaling was much lower than that of TCR signaling, both in Ca^2+^ flux (Figure 2A,B) and in the phosphorylation of downstream molecules (Figure 2C,D).

### 2.3. Distinct CAR Signaling Is Reflected by IL-2 Production Upon Activation by Antigen-Presenting Cells

**Figure 3:**
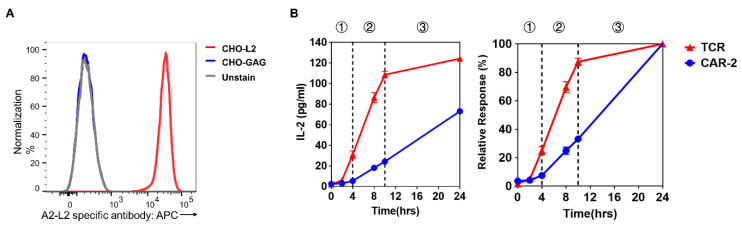
IL-2 production kinetics of CAR and TCR. (**A**) Expression of HLA*A02:01-L2 by CHO-APC. The TCR-like antibody was used to detect the expression and specificity. CHO-L2 and CHO-GAG refers to CHO-APC presented antigenic LMP2 A peptide and irrelevant GAG peptide, respectively. (**B**) IL-2 production of CAR-T and TCR-T upon stimulation by CHO-APC. The IL-2 production of TCR is separated by 3 phases, labeled as 1, 2, 3 in the graph, representing the acceleration, stable, and deceleration phases, respectively. The amount of IL-2 production in the left panel is calculated as a percentage and shown in the right panel. Data are representative of at least three independent experiments, plotted as mean ± SD of technical triplicates.

IL-2 production represents a downstream result of TCR signaling. We therefore tested and identified the difference in IL-2 production patterns between CAR-Jurkat and TCR-Jurkat cells. To this end, an artificial antigen presenting cell (APC) was constructed based on Chinese hamster ovary (CHO) cells. A single chain version of HLA-A*02:01 tethered to the L2 peptide was generated (Figure S4A). The transduced CHO-APC expressed the antigenic peptide-MHC (pMHC) complex, which was specifically recognized by the anti-HLA-A*02:01-L2 TCR-like Ab (Figure 3A). The scFv version of this TCR-like Ab was also shown to specifically bind to CHO cells expressing HLA-A*02:01-L2 (Figure S4B). The CAR-T and TCR-T cells were then incubated with the CHO-APCs. As seen in Figure 3B, TCR-T cells produced IL-2 in 3 phases within 24 h, with acceleration, stable, and then deceleration phases. The first phase started from 0 to 4 h, the second from 4 to 10 h, then the third from 10 to 24 h. In marked distinction, the CAR-T cells produced IL-2 only in a stable and consistent manner, in agreement with our previous observations, shown in Figure 2. Around 90% of the IL-2 production by TCR-Jurkat cells was finished by 10 h, yet only around 35% of IL-2 of CAR-T cells were produced by that timepoint (Figure 3B).

### 2.4. CD8 Coreceptor Is Recruited by TCR-Like CAR but Does Not Enhance Activation

**Figure 4:**
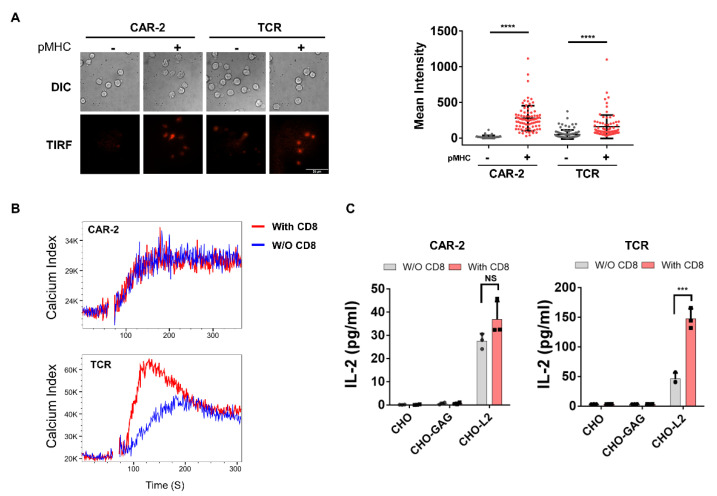
Interaction of TCR-like CAR CD8 coreceptor. (**A**) CD8 recruitment using TIRFM visualizing CD8α-mCherry. Left: representative images, scale bar is at 50 µm. Right: background-corrected mean fluorescence intensity of cell contact surface (*n* > 80 cells). (**B**) Ca^2+^ flux of CAR-T and TCR-T ± CD8. (**C**) CAR and TCR responsiveness ± CD8 to antigenic CHO-L2 or irrelevant CHO-GAG. Data are representative of at least three independent experiments, plotted as mean ± SD of technical triplicates. *** *p* < 0.001, **** *p* < 0.0001; NS: not significant at *p* > 0.05, by Student’s *t*-test.

Since L2-specific TCR-like CAR has similar specificity for pMHC as does the TCR, we tested the contribution of the CD8 coreceptor to CAR-T cell activation. CD8α was expressed as a fusion with the fluorescent protein mCherry (Figure S5A). We first showed that CD8 could be recruited to the immunological synapse (IS) by CAR-T cells, as is found in TCR-T cells [19,20], using total internal reflection fluorescence microscopy (TIRFM) to detect events at the contact surface (Figure 4A). A supported lipid bilayer with ICAM1, with or without pMHC was made. CAR-T or TCR-T cells were added onto the bilayer for 10 min. Mean fluorescence intensity (MFI) at the IS significantly increased in both CAR-T and TCR-T cells on bilayers containing pMHC, with clear clustering of CD8α-mCherry (Figure 4B, Figure S5B). We also calculated the MFI ratio inside/outside of the IS upon interaction between CAR-T or TCR-T cells with CHO-L2 cells using widefield fluorescent microscopy (Figure S5C). No significant difference was detected between CAR-T and TCR-T with CD8α-mCherry upon contact with CHO-APC, and both MFI ratios exceeded our cut-off ratio definition for IS formation of 1.5. These data show that that CD8α-mCherry was recruited in both CAR-T and TCR-T if cognate pMHC was present.

We next sought to identify whether CD8 would contribute to CAR-T functionally, since it was recruited to the CAR-T cell IS. After CD8α and β were co-transduced into both CAR-T and TCR-T cells, and expressions were shown to be similar (Figure S5D,E), reactivity of TCR-T bearing the CD8αβ coreceptor was significantly increased. As expected, CD8^+^ TCR-T showed faster and stronger Ca^2+^ flux and IL-2 production was nearly 3-fold higher than CD8^–^ TCR-T (Figure 4B,C). However, reactivity of CAR-T-bearing CD8 was not enhanced over CD8^–^ CAR-T. These data show that CD8 does not enhance CAR signaling, even though the CAR-recognized pMHC and CD8 were recruited to the IS of CAR-T cells.

## 3. Material and Methods

### 3.1. Plasmids and Sequences

The lentiviral vector, pLv, and its associated packaging plasmids, bearing Gag/Pol, Rev and VSV-G, respectively, were purchased from Vectorbuilder (Chicago, IL, USA). Chimeric antigen receptors with or without CD28 costimulatory sequences were synthesized and cloned into a lentiviral vector by Vectorbuilder. Human *CD8 A* and *CD8 B* genes were cloned from a human cDNA library in-house. The scFv construct for TCR-like Abs [17] was produced in P.A.M.’s lab (National University of Singapore, Singapore), and TCR construct specific for HLA-A*02:01 with peptide LMP2 A_426–434_ (from EBV) was a generous gift from Hans Stauss (University College London, London, United Kingdom). Single-chain trimer HLA-A*02:01-GAG was a gift from Keith Gould (Imperial College London, London, United Kingdom). Peptide mutagenesis: GAG (SLYNTVATL) to LMP2 A_426–434_ (“L2”: CLGGLLTMV) was done by using a Q5 mutagenesis Kit (New England Biolabs, Ipswich, MA, USA). All the molecular cloning work was done by using an In-Fusion HD cloning kit (Clontech, Mountain View, CA, USA), and single-chain trimer MHC constructs were cloned into pLv to generate artificial antigen-presenting CHO cells [21,22,23].

### 3.2. Cell Lines and Cell Culture

Endogenous TCR and coreceptor-deficient Jurkat76 was a kind gift from Heemskerk et al. [24]. Jurkat cells were maintained in complement RPMI-1640 media (Hyclone, Marlborough, MA, USA), supplemented with 10% fetal bovine serum (Hyclone), 2 mM L-glutamine (Gibco, Waltham, MA, USA), and MEM non-essential amino acid (Gibco) in a humidified 5% CO_2_ incubator at 37 °C. Human embryonic kidney epithelial cells (HEK293) were cultured in complete DMEM (Hyclone), which was supplemented with 10% fetal bovine serum (Hyclone), 2 mM L-glutamine (Gibco), and MEM non-essential amino acids (Gibco). The tetracycline-regulated expression (T-REx) CHO cell line was purchased from Invitrogen (Waltham, MA, USA) and used for the generation of artificial APC, which were single-cell sorted after transfection with single-chain MHC construct (scMHC). The CHO and CHO-APC cells were cultured in complete Ham’s F-12 (Gibco) medium, supplemented with 10% fetal bovine serum (Hyclone) and MEM non-essential amino acid (Gibco). The pMHC complex expression was checked regularly by flow cytometry.

### 3.3. Antibodies and Chemicals

The following antibodies were used in this study: Myc-Tag mouse mAb Alexa Fluor 647 (9 B11), anti-p44/42 ERK1/2, anti-ERK1/2 (all from Cell Signaling Technology, Danvers, MA, USA); anti-human CD3 APC and anti-B2 M APC (purchased from Biolegend, San Diego, CA, USA); anti-human CD8 A APC and anti-human CD8 B PE-Cy7 (all from eBioscience, San Diego, CA, USA); goat anti-mouse IgG (H  +  L) secondary antibody Alexa Fluor 647 (Thermo Fisher Scientific, Waltham, MA, USA); anti-Myc-Tag clone 4 A6 (Millipore, Burlington, MA, USA) was used for Western blotting; and specific HLA-A*02:01-L2 TCR-like antibodies were produced as described [17]. The scFv was produced by BL21 (DE3) (Novagen, Burlington, MA, USA) at 30 °C overnight, and the protein expression was induced by IPTG when OD_600_ reached 0.7. The bacteria were then pelleted and ultrasound sonicated. The scFv was purified through Ni-NTA beads (Thermo Fisher Scientific) and AKTA FPLC (GE) by size exclusion column and ion exchange column (MonoS, Sigma-Aldrich, St. Louis, MI, USA).

### 3.4. Lentivirus Production and Transduction

A total of 6.5 × 10^5^ HEK293 cells per well were seeded onto 6-well plates one day before transfection and incubated at 37 °C with 5% CO_2_. The cells were then transfected with packaging plasmid and lentiviral vector using polyethylenimine (PEI), and the medium was replaced after 12 h. The viral supernatant was harvested twice in the following two days. The collected viral supernatant was titered, filtered by a 0.45 mm membrane filter (Millipore), and concentrated by a 100 K ultracentrifuge tube (Millipore). For lentivirus transductions, polybrene and HEPES were added at 8–10 mg/mL and 10 mM, respectively, with Jurkat cells at 1 × 10^6^ per well in 1 mL, followed by spinoculation at 1200× *g*, for 2 h. For CHO cell transduction, the viral solution was directly added with the cell without spinoculation. After 24 h, cells and viral solutions were separated, and cells were cultured in the maintenance medium. After an additional 48 h culture period, flow cytometry analysis was performed to check the expression of the constructs.

### 3.5. Calcium Flux Assay

The Ca^2+^ flux assay was performed as previously described [25]. In brief, cell samples were suspended at a density of 10^7^ cells per ml in phosphate-buffered saline (PBS) and loaded with 2 mM Indo-1 AM (acetoxymethyl) for 30 min at 37 °C, followed by washing twice with culture RPMI. Cells were pre-warmed to 37 °C for 10 min before analysis and were kept at 37 °C in culture RPMI during the event collection. For cell stimulation, an HLA-A*02:01-L2 monomer was pre-refolded, biotinylated [26], and crosslinked with streptavidin Alexa 647 (Thermo Fisher Scientific) to form the antigen tetramer. Cells were then stimulated with monomers or tetramers. The mean fluorescence ratio of Indo-1 high to Indo-1 low was calculated using FlowJo (Version 10) kinetics program.

### 3.6. Western Blotting and Immunoprecipitation

A total of 10^6^ cells were lysed by NP-40 lysis buffer. Cell debris was pelleted at 13,000× *g* for 15 min at 4 °C, and the supernatant was collected and heated with reduced protein loading buffer (Nacalai-Tesque, Kyoto, Japan) or nonreduced loading buffer (without DTT). To detect phosphorylation after stimulation, 10^5^ CHO-APC cells were seeded per well in a 12-well plate one day before stimulation. Then, 10^6^ CAR-T or TCR-T cells were added into each well and incubated at 37 °C and 5% CO_2_ for the designated duration. The stimulated CAR-T or TCR-T cells were collected and prepared as above. All samples were loaded in a 4–12% Bis-Tris gradient gel (NuPAGE, Invitrogen, Waltham, MA, USA) and transferred to a PVDF membrane (Immobilon-FL Transfer Membrane, Millipore). The membrane was then blocked using blocking buffer (Odyssey, LI-COR, Lincoln, NE, USA) for 1 h at room temperature. Subsequently, the membrane was probed with different primary antibodies. The secondary antibodies used were IRDye 800 CW Goat anti-Mouse (926–32210, LI-COR) and IRDye 680 LT Goat anti-Rabbit (926–68021, LI-COR). Visualization and quantification of the blot was by the LI-COR Odyssey infrared imaging system.

### 3.7. T Cell Stimulation and Cytokine Secretion Assay

This was performed largely as described [22,23]. Artificial antigen presenting CHO cells (CHO-APC) were seeded onto a 96-well, flat-bottom plate at 2–3 × 10^4^ per well one day before the assay. Each CAR-T or TCR T cell sample was counted and suspended at a concentration of 10^6^ per mL in culturing RPMI medium. Then, 200 mL per well of suspended cell solution was added into the APC-CHO pre-seeded plate. Technical triplicates were performed for all experiments. The cells were incubated at 37 °C and 5% CO_2_ for 18 h or to designated timepoints. After incubation, the supernatant was collected for human IL-2 ELISA assay, which was performed according to the manufacturer’s protocol (Invitrogen).

### 3.8. Imaging

For total internal reflection fluorescence microscopy (TIRFM), lipid bilayers containing specific pMHC and other anchoring proteins were prepared as previously described [22,27]. In short, 0.2 mol% liposomes were used, evaporated by N_2_ at 37  °C and sonicated to prepare 4 mM lipid stock. Before adding the lipid, 6 M NaOH was used to clean glass 8-well chamber LabTekII chamber slides (Thermo Fisher Scientific) for 2 h and then they were rinsed with distilled H_2_O. The 4 mM lipid stock was diluted 10 times in PBS then added to the cleaned chamber slides and incubated for 30 min at room temperature. Then, 12 mL PBS was applied to wash away excess liposomes after incubation, and 2 mg/mL BSA was added to block the bilayers for 30 min, followed by the addition of 5 μg/mL streptavidin and incubation for 30 min. Biotinylated HLA-A*02:01-L2 monomer (produced as previously described [26]), recombinant human ICAM1 Protein, and hIgG1-Fc.His Tag (Thermo Fisher Scientific), after another wash for excess streptavidin, were added to the bilayers for 30 min. After washing, the bilayers loaded with specific pMHC were ready to be used. We prepared 10^6^ cells per mL of CAR-Jurkat or TCR-Jurkat with CD8α-mCherry and 100 mL of cells were added onto one well of the chamber at 37 °C for 10 min. Then, 100 μL of 8% paraformaldehyde was directly added to fix the cell and stop the stimulation. TIRF microscopy was later performed on an Olympus IX83 inverted microscope fitted with a four-laser TIRF module. Images were acquired using a 100×/1.49 NA oil-immersion lens. Fluorescence excited within the 100-nm evanescent field was recorded with a Hamamatsu ORCA Flash 4.0 camera. For regular fluorescent imaging, 10^5^ cells of CAR-Jurkat or TCR-Jurkat with CD8α-mCherry were mixed with 10^5^ cells specific CHO-APC in 100 μL RPMI medium for 10 min on one well of the chamber, followed by adding 100 μL of 8% paraformaldehyde. CD8 recruitment was later detected on an Olympus IX83 inverted microscope in the normal module. Images were acquired using a 20–40×/1.49 NA oil-immersion lens.

### 3.9. Flow Cytometry and Cell Sorting

BD LSR Fortessa X-20 (Becton Dickinson, Franklin Lakes, NJ, USA) was regularly used to detect the expression of the protein transduced and for conducting flow cytometry experiments. Mo-Flo XDP (Beckman Coulter, Inc., Brea, CA, USA) or SY3200 (Sony Biotechnology Inc., San Jose, CA, USA), in the Flow Cytometry Laboratory, Immunology Program, National University of Singapore, were used to purify transduced cells and perform single-cell sorting. All flow data analysis was performed in FlowJo (Version 10).

### 3.10. Statistical Information and Data Analysis

The two-tailed Student’s *t*-test was used to test the significance between different sample groups throughout the study in GraphPad Prism (Version 7). Variance was considered similar in different sample groups. The significance cut-off (*p* value) was 0.05.

## 4. Discussion

TCR signaling has been studied for several decades. Though incompletely understood, three main mechanisms have been proposed regarding how TCR signaling is triggered: receptor clustering; mechanosensing, in which the TCR complex experiences a conformational change [28]; and size-dependent protein segregation, where large cell surface proteins, including the phosphatase CD45, are excluded so that phosphorylation is then triggered by kinases [29]. We took advantage of the ability to make pMHC molecules into monomeric or tetrameric forms to study the receptor clustering effects of CAR signal triggering. Compared with antigens presented by cells [30], soluble antigens will not form immunological synapses. Therefore, the soluble pMHC, either monomeric or tetrameric form, would provide a better way to study the cluster effect for the triggering of signals at the molecular level. We found that only the tetrameric form of the antigen was able to trigger CAR signaling, but not the monomeric form, even though pMHC monomer had been shown to bind strongly to the CAR. This indicated that receptor clustering is important for CAR signal triggering. The results are consistent with other findings using dimerized GFP and TGF-b as the antigens for CAR [31]. On the other hand, TCR also showed a requirement for antigen clustering to trigger the signal. However, monomeric antigen has been demonstrated as sufficient to initiate TCR signaling [32,33]. The disparate observations could be due to other signaling molecules such as the CD8 coreceptor, which were deliberately not involved in this model, that has been shown to facilitate monomer activation for TCR [34]. Therefore, without facilitation from these molecules, oligomerization would still be important for TCR signal triggering. In our experiments, the tetramer formed through the interaction between biotin and streptavidin was not homogenous, as shown in the supplemental data. CAR-1 and CAR-2 were also detected to be dimers bound by covalent disulfate bonds. To what degree the CAR needs to be clustered is not known under this circumstance. However, a study done by Chang et al. suggests that a dimeric form of the antigen would suffice to trigger CAR signaling [31].

While Ca^2+^ flux was activated by the pMHC tetramer, we observed distinct signaling kinetics between CAR and TCR, where CAR seemed to sustain the Ca^2+^ flux but TCR signaling waned after quickly reaching a peak. To our surprise, the Ca^2+^ flux of CAR continued even 10 min after activation. The strength of the sustained CAR signal was much weaker than the peak of TCR signal, but close to that of TCR signal after 10 min. To rule out the impact of the costimulatory domain of the second generation CAR, we demonstrated, by using the first generation CAR, that the sustained CAR signaling could be attributed to the basic CAR design (the recognition the scFv domain, stalk, transmembrane region, and CD3 z domains) and not to the costimulatory domain. Besides, the activation curves shown by TCR and CAR are reminiscent of the binding profiles of TCR and the antibody tested by surface plasmon resonance (SPR), where the antibody has a dramatically longer K_off_ than that of TCR [17]. This similarity may infer that it is the affinity profile of each recognition domain, i.e., scFv or the variable domains of TCR, that makes their signaling kinetics distinct. It is plausible that as long as CAR is conjugated with an antigen and clustered, the signal activation would last. The prolonged and high affinity contact with the surface antigen may also contribute to trogocytosis, which contributes to the antigen escape of cancer cells [8]. Another point worth noting is the lower magnitude of CAR signaling compared to that of TCR signaling. This may be due to each TCR complex comprising 6 CD3 subunits with 10 immunoreceptor tyrosine-based activation motifs (ITAMs), whereas each CAR only has 3 ITAMS. Thus, CAR activates a long but low downstream signal contrast with TCR, which activates a short but high downstream signal.

IL-2 production represents a culmination of signaling cascades, requiring activation of all three signaling pathways downstream of TCR signaling [35]. We examined the distinct signal kinetics based on IL-2 production. Consistent with the sustained CAR signaling detected, we observed that IL-2 production by CAR-T cells is more stable than that of TCR where TCR produced IL-2 in three phases within 18 h, but CAR-T cells produced IL-2 in a relatively linear manner. This CAR signaling pattern may also potentially explain why CAR-T cells could activate the GSDME signaling pathway of cancer cells, since CAR activation could be accumulated in the long-term, leading to enhanced killing functions.

We investigated a CAR with TCR-like specificity to enable comparison to conventional TCR and to test whether certain canonical signaling molecules, such as CD8, are involved in CAR signaling. We tested whether TCR-like CAR would use assistance from the CD8 coreceptor to enhance CAR-T activation, in a similar manner to that of TCR. However, no significant augmentation of either immediate calcium flux or later IL-2 production was observed, even though we found CD8 recruitment to the IS on CAR-T. We previously showed that CD8 can be concentrated into the IS, so long as MHC-I is available on the APC, even in the absence of TCR [19,20]. The CD8 coreceptor enhances the sensitivity of TCR through its interaction with LCK and stabilizes its binding with the antigenic pMHC complex [36] or non-antigenic, co-agonist pMHCs [21,22]. The inability of the CD8 coreceptor to enhance CAR signaling could potentially be explained by extracellular and intracellular effects. Since CAR has a significantly higher affinity than that of TCR, the stabilization effects from CD8 to TCR may not be required for CAR, which have been partly demonstrated by its capacity for monomer binding. On the other hand, because CD8 binds to a non-polymorphic part of the MHC-I a3 domain, and TCR binds in a conserved orientation to the pMHC, it ensures that the CD8-associated LCK is recruited in a particular orientation to the complex of TCR-CD3 and pMHC [37]. The orientation of the TCR-like CAR recognizing pMHC-I is not subject to such constraints, so that CD8 recruiting LCK to the pMHC is unlikely to force it into a suitable orientation to start signaling through the CD3 z subunit of the CAR. Intracellularly, LCK is better able to bind and phosphorylate CD3 e rather than CD3 z to initiate the cascade [38], so this may also be a factor in the lack of a CD8 requirement to initiate the CAR signaling.

In our study, we compared CAR with TCR signaling using Jurkat T cells. Although studies on Jurkat T cells have produced many seminal findings on T cell signaling [39], using Jurkat as a model system has limitations. Jurkat cells are deficient in tumor suppressor phosphatase PTEN, which dephosphorylates phosphatidylinositol (3,4,5)-trisphosphate (PIP3) to phosphatidylinositol (4,5)-trisphosphate PIP2. This deficiency causes a constitutive activation of AKT [40,41]. The study we report here involves early signal triggering and extracellular molecular interactions, which are not directly involved in PTEN-AKT signaling, suggesting that similar results would be obtained in primary cells, although this remains to be directly demonstrated.

## 5. Conclusions

We constructed a CAR with the same specificity as the TCR construct targeting peptide-MHC with the aim to understand the CAR signal triggering and activation patterns. CAR signal triggering was demonstrated to require receptor clustering. In addition, CAR transduces the signaling in a more sustained manner while in a lower magnitude compared with that of TCR. Furthermore, the CD8 coreceptors, though recruited by TCR-like CAR, play a limited role in enhancing the CAR signaling, at least for CD28-CAR.

## Data Availability

The datasets generated during and/or analyzed during the current study are available from the corresponding author on request. The data are not publicly available due to no relevant data to upload to a database.

## Supplementary Materials

The following are available online at https://www.mdpi.com/2072-6694/13/4/867/s1, Figure S1: CAR constructs and pMHC tetramer, Figure S2: Ca^2+^ flux of first generation CAR in long-term activation, Figure S3: Uncropped Western Blots of Figure 2C and Figure S1, Figure S4: Artificial CHO antigen-presenting cells, Figure S5: CD8 recruitment to the IS and functional impact of CD8 for CAR-T and TCR-T.
