# Supplementary material for: Targeting CAR to the Peptide-MHC Complex Reveals Distinct Signaling Compared to That of TCR in a Jurkat T Cell Model
Source: Cancers (Basel). 2021 Feb 18;13(4):867. doi: 10.3390/cancers13040867 (PMC7922477; doi:10.3390/cancers13040867)
Supplement: Supplementary file 1 [file cancers-13-00867-s001.pdf]

cancers

MDPI

Supplementary Material: Targeting CAR to the Peptide-MHC Complex Reveals Distinct Signaling Compared to That of TCR in A Jurkat T Cell Model

Ling Wu, Joanna Brzostek, Shvetha Sankaran, Qianru Wei, Jiawei Yap, Triscilla Y.Y. Tan, Junyun Lai, Paul A. MacAry and Nicholas R. J. Gascoigne

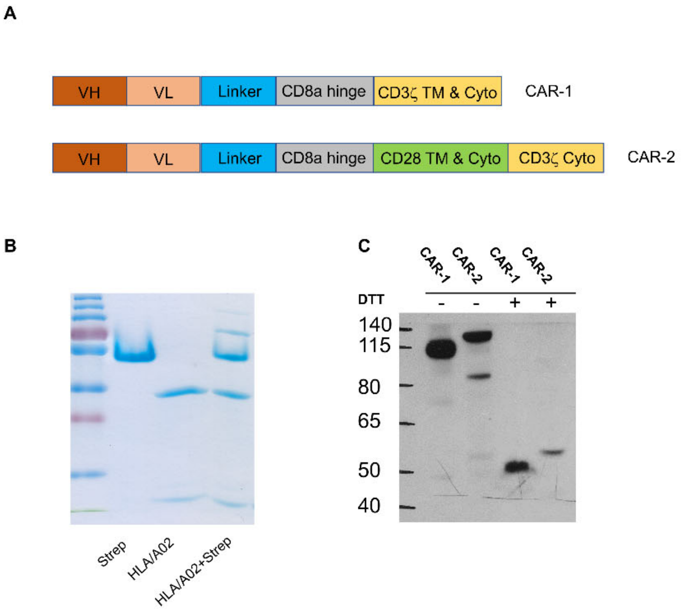

Figure S1. CAR constructs and pMHC tetramer. (**A**) graphic presentation of first and second generation of CAR design. The linker region contains Myc-tag for detection of CAR expression. (**B**) Formation of pMHC tetramer. The coomassie blue gel shows the molecular size change after biotinylated monomer incubating with streptavidin. (**C**) Clustering of CAR-1 and CAR-2 detected in nonreduction electrophoresis. The protein sizes calculated for each lane from left to right are 107 kDa, 125 kDa, 50 kDa, and 54 kDa respectively.

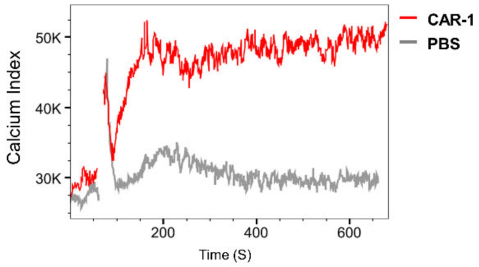

Figure S2. Calcium flux of first generation CAR in long term activation.

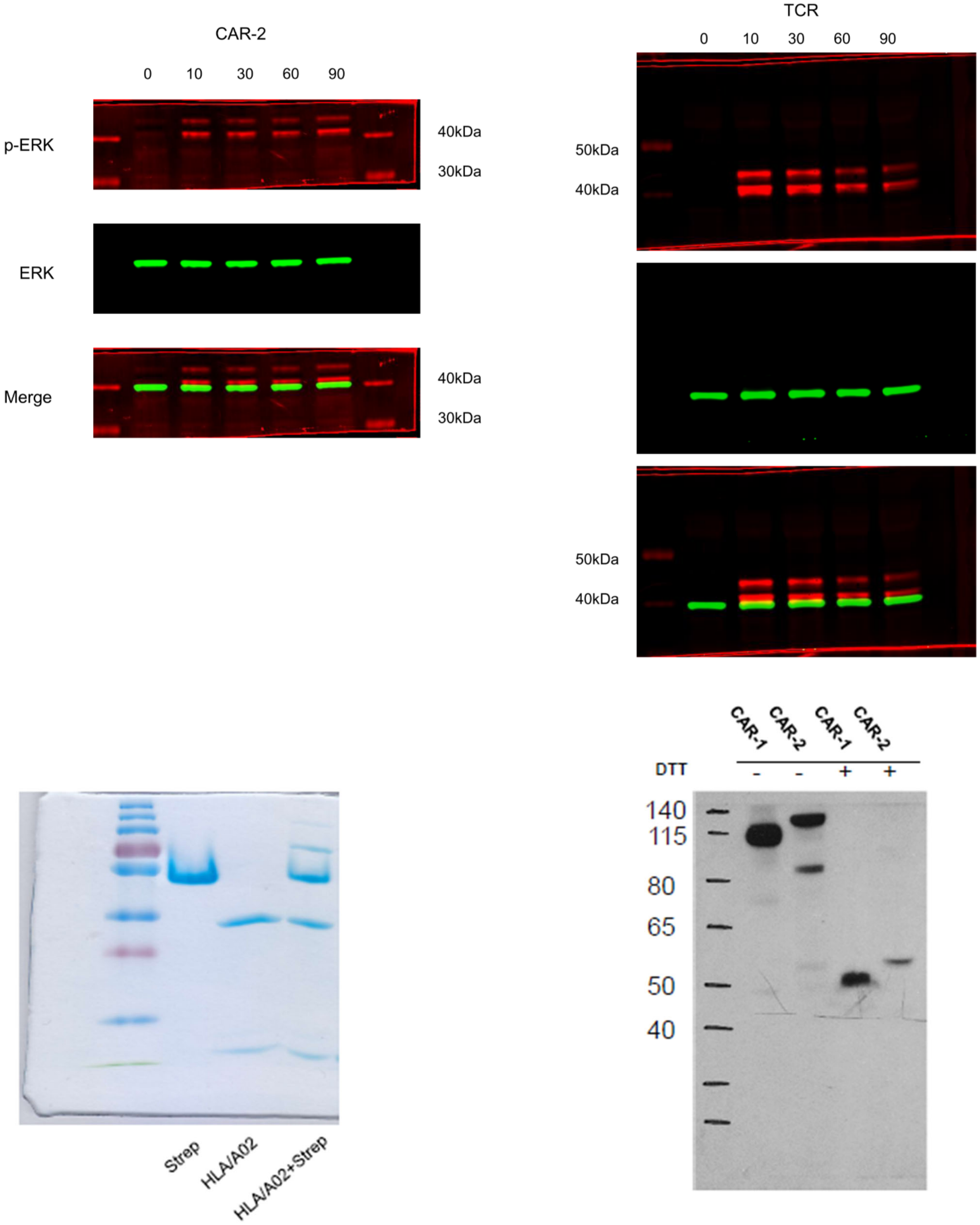

Figure S3. Uncropped Western Blots of Figures 2C and S1.

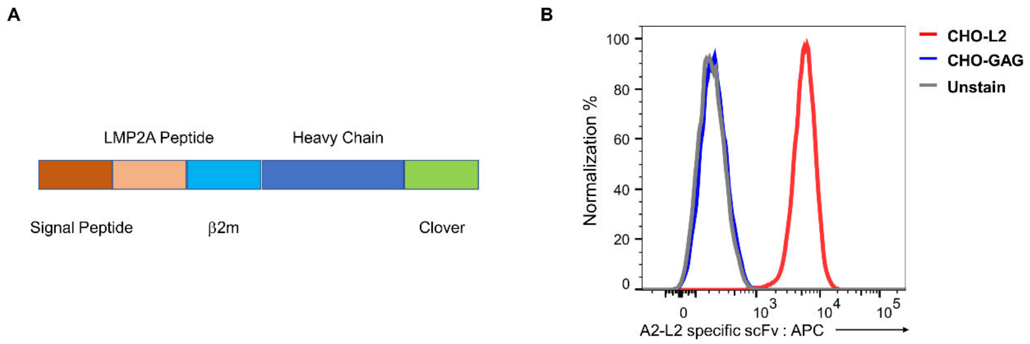

Figure S4. Artificial CHO antigen presenting cells. (**A**) Schematic diagram of the single-chain HLA (scHLA) construct with linked peptide. (**B**) The CHO-APC stained with scFv from specific TCR-like antibody.

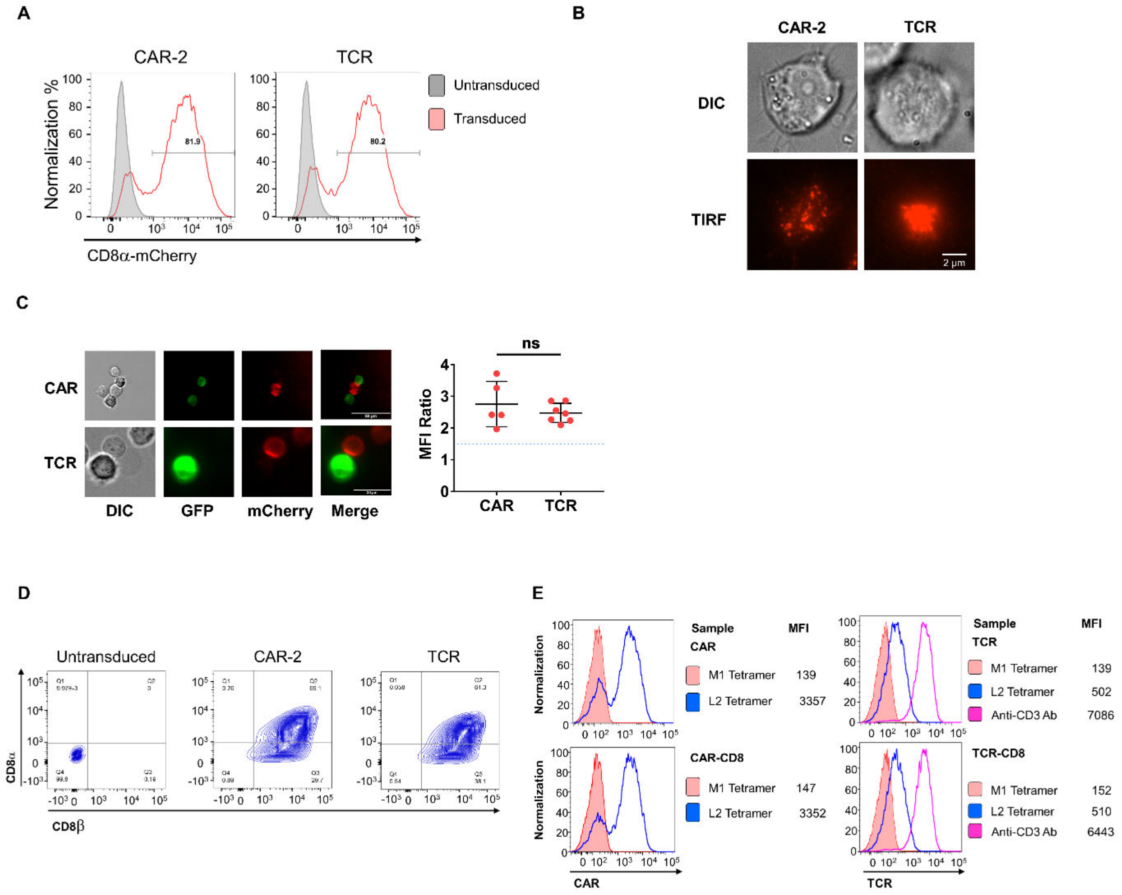

Figure S5. CD8 recruitment to the IS and functional impact of CD8 for CAR-T and TCR-T. (**A**) CD8α-mCherry transduction in CAR-T and TCR-T; Scale Bar: 2μm. (**B**) Single cell image zoom in from TIRF microscope. The image is a representative of CD8α-mCherry expressed CAR-T and TCR-T cells analyzed by TIRF microscope; Scale Bar: 2 μm. The cells were activated by antigenic pMHC, HLA-A*02:01-L2, presented by lipid bilayer. (**C**) CD8 recruitment to the IS analysed using fluorescence microscopy; Scale Bar: top 50 μm, below, 20 μm. The left panel is a representative image graph (n ≥ 5), GFP channel detected the presence of CHO-APC expressing HLA-L2, and mCherry detected the localization of CD8α-mCherry. The right panel shows quantified data of the mean fluorescent intensity (MFI) ratio, which was calculated by mCherry MFI of the IS to that of membrane outside IS. Cut-off value (1.5) is marked by a blue dashed line. Data are plotted as mean ± SD, n ≥ 5 cell conjugates (ns, $p > 0.05$). (**D**) Co-transduction of CD8α and CD8β construct on CAR-Jurkat or TCR-Jurkat cell. CD8α and CD8β construct were on different lentiviruses and co-transduced in CAR or TCR-Jurkat cells. (**E**)

CAR and TCR expression after CD8α and CD8αβ co-transduction. The HLA-A*02:01-L2 tetramer was used for CAR-T and TCR-T staining. TCR expression was also detected by the anti-CD3 antibody. The total MFI is shown on the right side of each graph.
